# Implementing Alternatives to Coercion in Mental Health Care

**DOI:** 10.1192/j.eurpsy.2022.35

**Published:** 2022-09-01

**Authors:** S. Galderisi

**Affiliations:** University of Campania Luigi Vanvitelli, Department Of Psychiatry, Naples, Italy

## Abstract

**Abstract Body:**

The use of coercive measures in Medicine represents a controversial issue. Even when they comply with all rules and procedures and are enacted with the intention to address the health needs of the patient, and/or protect the patient and/or others, they always represent an infringement of fundamental personal rights and require strong ethical justification.

In Psychiatry the debate around coercive measures has led to a theoretical impasse, as the attempt to solve an ethical dilemma may expose mental health care to other ethical challenges and questions of competing rights. At the same time, the ongoing debate has contributed to raise the awareness that coercive practices are over-used, and mental health care is in need of a profound transformation towards recovery-oriented systems of care.

The implementation and dissemination of alternatives to coercive practices is an essential component of such transformation. Relevant research has provided tools and documented successful practices, and initiatives aimed at making these resources available and adapted to different contexts are being promoted by international organizations, professional associations and associations of users and carers.^1^

The profound transformation of current mental health care towards recovery-oriented systems of care requires resources and shared goals among the different stakeholders. Integrated and personalised care pathways, respect of human rights, shared decision making, and involvement of users and carers are essential components of this transformation.

**Disclosure:**

No significant relationships.

